# Ferns: the missing link in shoot evolution and development

**DOI:** 10.3389/fpls.2015.00972

**Published:** 2015-11-06

**Authors:** Andrew R. G. Plackett, Verónica S. Di Stilio, Jane A. Langdale

**Affiliations:** ^1^Department of Plant Sciences, University of OxfordOxford, UK; ^2^Department of Biology, University of WashingtonSeattle, WA, USA

**Keywords:** plant, evolution, development, shoot, monilophyte, fern, *Ceratopteris*

## Abstract

Shoot development in land plants is a remarkably complex process that gives rise to an extreme diversity of forms. Our current understanding of shoot developmental mechanisms comes almost entirely from studies of angiosperms (flowering plants), the most recently diverged plant lineage. Shoot development in angiosperms is based around a layered multicellular apical meristem that produces lateral organs and/or secondary meristems from populations of founder cells at its periphery. In contrast, non-seed plant shoots develop from either single apical initials or from a small population of morphologically distinct apical cells. Although developmental and molecular information is becoming available for non-flowering plants, such as the model moss *Physcomitrella patens*, making valid comparisons between highly divergent lineages is extremely challenging. As sister group to the seed plants, the monilophytes (ferns and relatives) represent an excellent phylogenetic midpoint of comparison for unlocking the evolution of shoot developmental mechanisms, and recent technical advances have finally made transgenic analysis possible in the emerging model fern *Ceratopteris richardii*. This review compares and contrasts our current understanding of shoot development in different land plant lineages with the aim of highlighting the potential role that the fern *C. richardii* could play in shedding light on the evolution of underlying genetic regulatory mechanisms.

## Introduction

Land plants (embryophytes) evolved from aquatic green algae ∼470 million years ago, with phylogenetic analyses consistently positioning charophytic (streptophyte) algae as the closest extant sister group (Karol et al., 2001; Lewis and McCourt, 2004; Wodniok et al., 2011; Ruhfel et al., 2014). Whilst charophytes exhibit a range of vegetative body plans in the haploid (gametophyte) generation of the lifecycle (reviewed in Niklas and Kutschera, 2010), the diploid (sporophyte) generation of the lifecycle is unicellular; the single-celled product of gamete fusion (zygote) directly undergoes meiosis. By contrast, in all land plants the zygote undergoes intervening mitotic divisions to create a multicellular sporophyte (the embryo), the uppermost part of which has become specialized into a photosynthetic shoot. Although a multicellular sporophyte is the defining characteristic of land plants, the structure has undergone enormous diversification and elaboration during evolution, from simple and transient (as in the stalked sporangium in most bryophytes) to highly complex and long-lived (as in the tree-forms of various vascular plants). In all cases, however, meiosis ultimately generates haploid gametophytes to complete the lifecycle.

**FIGURE 1:**
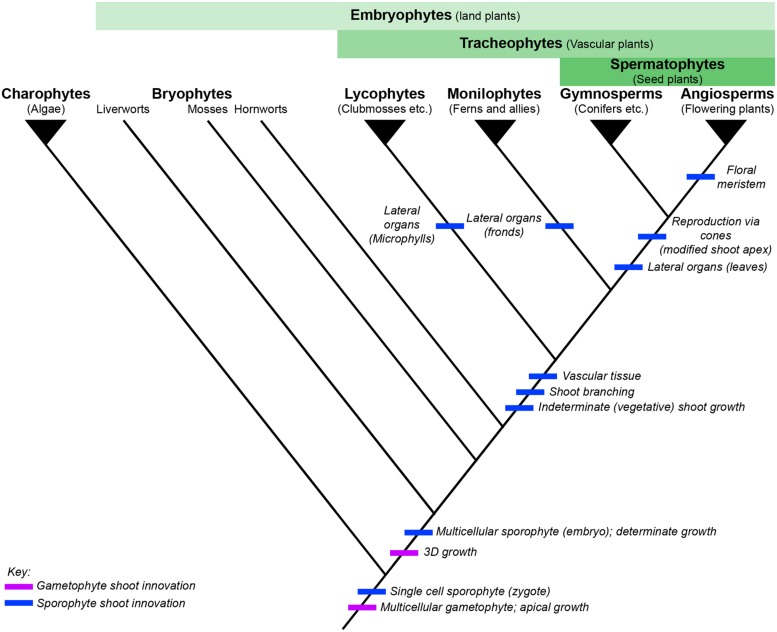
**The evolution of shoot development across land plants**. Simplified phylogenetic tree of extant land plants, based on Qiu et al. (2006), charophytic algae shown as outgroup. Filled triangles represent monophyletic clades, whereas the bryophyte grade is paraphyletic. Broader clades used for reference in this review are defined by the green bars above the phylogeny. Key innovations relating to shoot development are marked on the tree, relating to gametophyte shoot architecture (purple) or sporophyte shoot architecture (blue), respectively.

**FIGURE 2:**
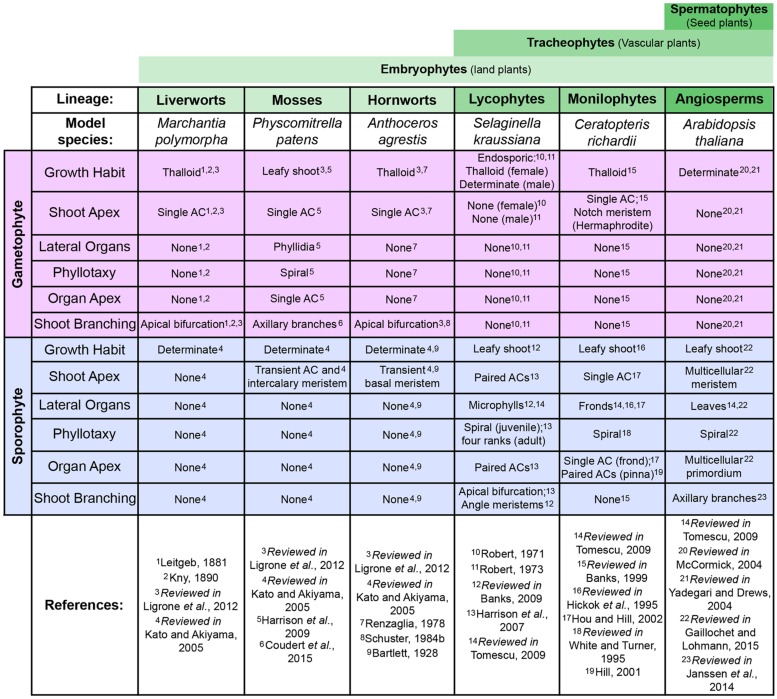
**Comparing shoot development across model land plants**. Comparison of shoot characteristics found in the gametophyte (pink) and sporophyte (blue) generations of select developmental/genetic model plant species, including examples from most extant land plant (embryophyte) lineages. Broader clade denominations between these lineages are indicated by green bars above (see **Figure 1**). It should be noted that not all model species are entirely representative of development within each lineage, in particular the mosses and liverworts. Examples from other species and fossil data are included in this review where necessary to provide a more accurate representation of evolutionary trajectories.

Successive land plant lineages have innovated new sporophytic shoot structures, leading to increasing morphological and physiological complexity (**Figure 1**). Understanding the genetic mechanisms underlying the origins and continued modification of the land plant shoot is one of the primary aims of research into plant evolution and development (evo-devo). Although the characterization of evolutionary trajectories is not always straightforward, because many lineages that contained informative intermediate characters are now extinct, reconstruction is possible through comparison of extant species to infer plesiomorphies (ancestral traits) and apomorphies (derived traits). Our understanding of how land plant morphologies evolved is based mostly on comparative developmental studies between representative model species, predominantly the flowering plants *Arabidopsis thaliana* and *Oryza sativa* and the moss *Physcomitrella patens*. Further models are increasingly being exploited as experimental systems for molecular analyses, including the liverwort *Marchantia polymorpha* and lycopods in the genus *Selaginella* (the genome of *Selaginella moellendorffii* has been sequenced (Banks et al., 2011) whilst the bulk of developmental data comes from *S. kraussiana*). A substantial amount of detailed developmental data has been accumulated in these and other non-seed plant lineages (reviews include White and Turner, 1995; Banks, 2009; Renzaglia et al., 2009; Ligrone et al., 2012; Vasco et al., 2013, and references therein). Key developmental characteristics relating to shoot development are summarized and compared between the different models discussed in this review in **Figure 2**. However, at present the bulk of gene function data available outside of the angiosperms is from the moss *P. patens*. The large evolutionary distance between these two ends of the land plant phylogeny has made interpretation of such comparisons extremely challenging and, in some cases, of little use. It should be noted that model species are not always wholly representative of their extant relatives, nor necessarily the ancestral state of that particular lineage. This is particularly problematic in bryophyte lineages such as the mosses, where the fossil evidence and diversity in extant species highlight the potential for confusion over the ancestral state (see Shoot Branching section). Although in this review we necessarily focus on the combined genetic and developmental data available from model species, where required we highlight fossil data or examples from non-model species to better represent evolutionary trajectories or the ambiguity currently surrounding them.

One group of plants that is notably absent in most comparative studies is the monilophytes (ferns and their relatives). Monilophytes are the most closely related extant land plant lineage to seed plants (**Figure 1**; Pryer et al., 2001). As such, monilophytes are a highly informative phylogenetic node, both as outgroup to the seed plants and as an intermediate lineage to provide resolution for functional comparisons between homologous genes in bryophytes and angiosperms. That said, the monilophytes themselves represent an ancient and highly diverse lineage, diverging from the seed plants 400 million years ago (Pryer et al., 2001) and encompassing a wide variety of growth habits including tree forms, aquatics and epiphytes (reviewed in Schuettpelz and Pryer, 2008; Watkins and Cardelús, 2012). The largest clade of ferns, the leptosporangiate ferns, account for approximately 80% of non-flowering vascular plant species (Schuettpelz and Pryer, 2009). A number of developmental innovations have occurred independently within the monilophyte lineage, including the evolution of lateral organs (fronds) and heterospory (**Figure 1**). However, to date our understanding of fern developmental genetics has been impeded by serious technical barriers that are only now being overcome. These barriers include typically very large genomes (Barker and Wolf, 2010; Bainard et al., 2011), an obstacle further complicated by frequent polyploidy (Wood et al., 2009), and a lack of any genetic transformation system. Two fern species are now coming to prominence as research vehicles: *Ceratopteris richardii*, a homosporous fern long-established in laboratories for developmental studies and teaching (Hickok et al., 1995); and the heterosporous aquatic fern *Azolla filiculoides*, a species potentially well-suited for industrial biomass production (Brouwer et al., 2014). Efforts are currently underway to sequence the genomes of both species (Sessa et al., 2014), and a wealth of transcriptome data is being generated in diverse fern species via the 1 KP project (Wickett et al., 2014). In addition, a number of stable genetic transformation methods have recently been reported, including methods that are suitable for *C. richardii* (Muthukumar et al., 2013; Plackett et al., 2014; Bui et al., 2015). In light of these advances, the study of ferns to aid our understanding of shoot evolution is being viewed with increasing enthusiasm (Bennett, 2014; Banks, 2015; Harrison, 2015). A review of our current understanding of the genetic regulation of shoot development across the land plants, including what little is already known about monilophytes, is thus timely and presents an opportunity to outline the key developmental questions that need to be answered.

## The Evolution of Land Plant Shoots

**FIGURE 3:**
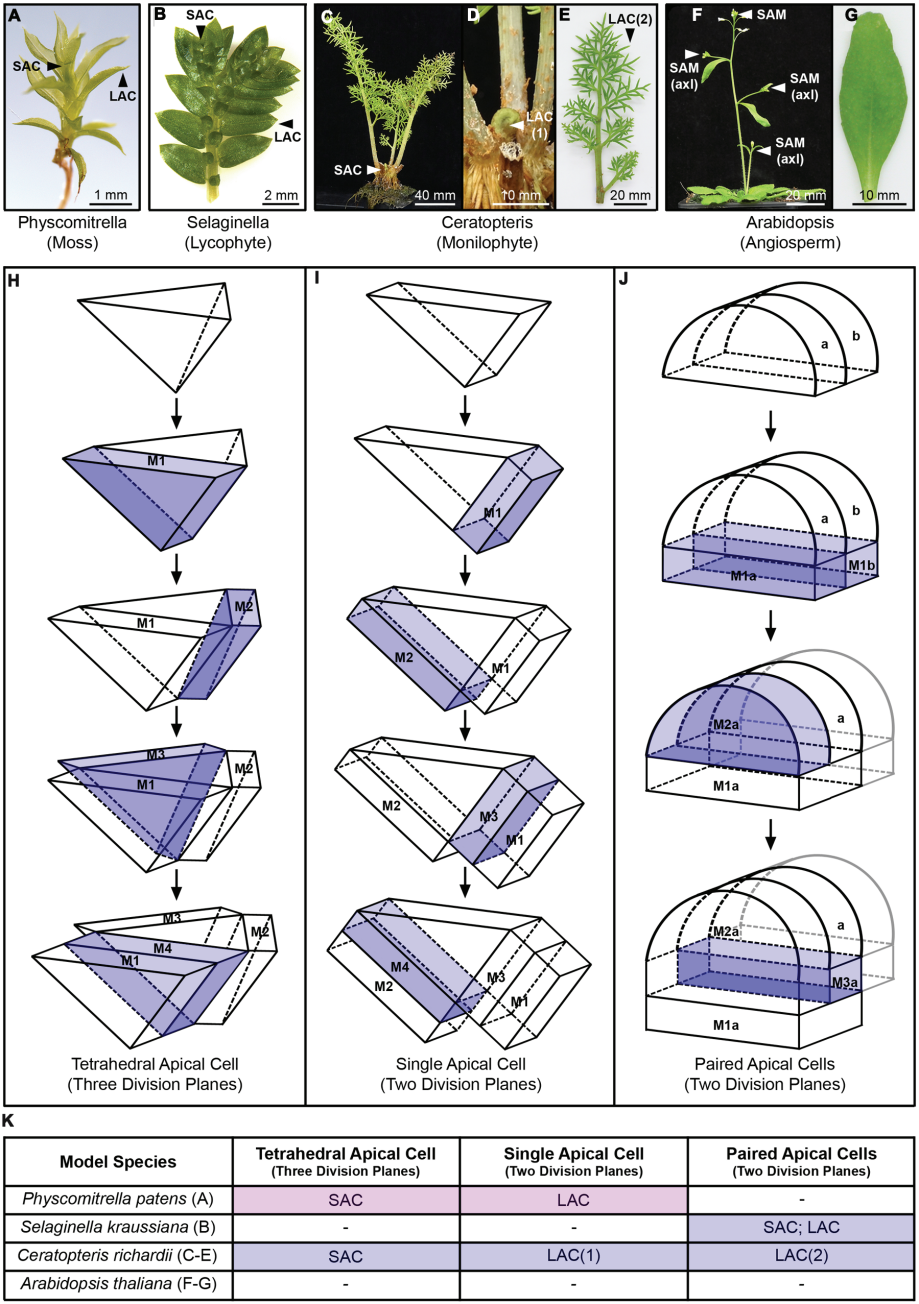
**Shoot apical activity across representative model land plants. (A–G)** Shoots and lateral organs of representative model land plant species; **(A)** *Physcomitrella patens* gametophore; **(B)** *Selaginella kraussiana* shoot and microphylls, showing unequal apical branching; **(C–E)** *Ceratopteris richardii* sporophyte **(C)**, showing emergence of new frond from the shoot apex **(D)** and a fully developed reproductive frond with lateral pinnae **(E)**; **(F,G)** *Arabidopsis thaliana* sporophyte with axillary branches emerging **(F)**, showing a rosette leaf **(G)**. The position of shoot apical cells (SAC) and lateral apical cells (LAC) are marked. *A. thaliana* develops from a multicellular shoot apical meristem (SAM), and axillary branches develop from the activity of similar, axillary meristems [SAM(axl)]. *A. thaliana* leaves develop from a multicellular primordium and lack an apical cell (AC) or meristem. **(H,I)** Diagrammatic summary of different AC geometries and division patterns. **(H)** tetrahedral AC with three cutting faces; **(I)** single AC with two cutting faces; **(J)** adjacent paired ACS with two cutting faces each. Daughter cells (merophytes), generated through asymmetric divisions that reconstitute the AC, are marked M, and numbered in order of their production. For clarity, the most recently formed merophyte is highlighted in blue. In the case of paired ACs, these and their descendants are distinguished by ‘a’ and ‘b’ accordingly. In the interests of clarity, beyond the first division only the further divisions of AC ‘a’ are shown: these are mirrored by the activity of AC ‘b’. The complex multicellular SAM of *A. thaliana* is not shown. **(K)** Table summarizing the developmental contexts in which the different shoot ACs in **(H–J)** are found, referring to the labels marked in **(A–G)** and distinguishing whether they occur in the gametophyte (pink) or sporophyte (blue) generation.

The alternation of multicellular haploid gametophyte and diploid sporophyte generations is a shared feature of all land plant lifecycles. However, the relative dominance of each generation changed as new land plant lineages evolved. In bryophytes (liverworts, mosses, and hornworts) the dominant generation of the lifecycle is the gametophyte. For example, the haploid spores of *P. patens* germinate to form filamentous gametophytes that transition into shoot-like structures (gametophores; **Figure 3A**) that produce leaf-like organs (phyllidia) and ultimately male and female gametangia (gamete-producing structures; reviewed in Kofuji and Hasebe, 2014). Upon fertilization, the diploid zygote undergoes a strictly determinate developmental program to become an unbranched sporophyte axis terminating in a single sporangium. Within vascular plants (tracheophytes) the role of the sporophyte generation increased at the expense of the gametophyte, which fossil evidence suggests occurred at the base of the clade (reviewed in Gerrienne and Gonez, 2011). Indeterminate branched sporophytes are found in all tracheophyte lineages, for example the lycophyte *S. kraussiana* (**Figure 3B**), whilst the *S. kraussiana* female and male gametophytes, respectively, produce a thallus inside the megaspore or directly generate gametangia upon spore germination (Robert, 1971, 1973). Similar development has been recorded in the related species *S. apoda* (Schulz et al., 2010). Gametophyte development in monilophytes is also reduced compared to bryophytes. The *C. richardii* gametophytes develop as a single cell-layered thallus comprising a few specialized cell types (reviewed in Banks, 1999). The subsequent sporophyte develops as an indeterminate shooting structure, producing fronds sequentially from a persistent post-embryonic shoot apex (**Figures 3C–E**; Johnson and Renzaglia, 2008). In angiosperms the sporophyte develops a highly complex, indeterminate body-plan from multiple post-embryonic shoot apical and axillary meristems (**Figure 3F**; Gifford and Foster, 1989), whereas the male and female gametophytes comprise just a few cells each (reviewed in McCormick, 2004; Yadegari and Drews, 2004). In bryophytes and angiosperms the sporophyte and gametophyte, respectively, are fully dependent on the dominant stage of the lifecycle for nutrition (matrotrophic), whereas in both lycophytes and ferns the gametophyte develops independently of the sporophyte and, beyond a transient period where the sporophyte embryo develops upon the gametophyte, the sporophyte is not nutritionally dependent upon the gametophyte (reviewed in Qiu et al., 2012).

From a developmental perspective, canonical plant shoots (as generally recognized in vascular plants) can be defined as a process, i.e., they develop iteratively from an apex to produce lateral organs. Using this definition, the gametophores of extant mosses and ‘leafy’ liverworts can also be classified as shoots, possessing an axial body-plan. In contrast other liverwort species (including *M. polymorpha*) and all hornworts develop a thalloid body-plan (comprising multiple cell layers), which possesses apical growth in common with shoots but lacks defined lateral organs (**Figure 2**; reviewed in Renzaglia et al., 2009; Ligrone et al., 2012; Villarreal and Renzaglia, 2015). Although presumably arising from a common origin, the precise evolutionary relationship between the axial and thalloid body-plans in early diverging embryophytes is not yet fully resolved (reviewed in Qiu et al., 2012), and so at present it is not possible to assess character polarity. It is, however, quite probable that shared developmental characters are underpinned by conserved genetic mechanisms (see examples given in the review below).

A second important component to this definition of the shoot is the concept of indeterminate growth. The *P. patens* sporophyte demonstrates apical growth but only transiently, terminating after just a few cell divisions in a sporangium (reviewed in Kato and Akiyama, 2005). Recent transcriptome data from developing liverwort and moss sporophytes indicates expression of meiosis-associated genes even during embryonic stages (Frank and Scanlon, 2015), further suggesting that these sporophytes lack indeterminacy and thus true shoot function. Similarly, the thalloid gametophytes of the ferns *C. richardii* and *Lygodium japonicum* initially grow from transient apical cells (ACs) that then terminate (Banks, 1999; Takahashi et al., 2015). Interestingly, in both species, growth of the chordate hermaphrodite thallus continues through proliferation of a second, distinct multicellular meristematic region (the ‘notch meristem’), iteratively generating archegonia until successful fertilization has occurred (Banks, 1999; Takahashi et al., 2015). Development of the strap-like thalloid gametophyte of the epiphytic fern *Colysis decurrens* follows the same principles, with a single transient early AC followed by an indeterminate multicellular marginal meristem (Takahashi et al., 2009). The parallels with canonical shoot development are striking, but whether the notch/marginal meristem represents the reduction of an ancestral gametophytic shoot has yet to be determined.

The origins and early evolutionary trajectory of the vascular plant shoot are much debated, with several competing theories presented (reviewed in Tomescu et al., 2014), but there is general agreement on the key developmental innovations that occurred during shoot evolution: indeterminate apical activity, organogenesis, shoot branching, and developmental phase-change.

## Shoot Indeterminacy- Apical Cells Versus Multicellular Meristems

Cells with shoot apical function (i.e., having indeterminate cell fate) are present in all extant land plant lineages (**Figure 2**; Steeves and Sussex, 1989). In seed plants (gymnosperms and angiosperms) these are part of a highly organized, multicellular shoot apical meristem (SAM), whereas in non-seed plants (bryophytes, lycophytes, and monilophytes) they exist as a distinct single AC, or small cluster thereof (**Figures 2** and **3**). Although they vary in size, shape, and number of cutting planes, ACs can be defined as dividing asymmetrically to produce derivatives and replenish themselves.

The *P. patens* gametophore possesses a single persistent tetrahedral (pyramid-shaped) AC that cleaves sequentially in three planes to generate determinate leaf-like organs (**Figure 3H**; Harrison et al., 2009). Tetrahedral ACs are also found in the gametophytic shoots produced by ‘leafy’ liverworts (Crandall-Stotler, 1980) but these undergo a different pattern of asymmetric cell division that could indicate convergent evolutionary origins, a suggestion supported by differing formative division planes during lateral organ formation (Crandall-Stotler, 1986). In contrast, the single ACs of thalloid bryophyte gametophytes display a different geometry, at maturity cleaving across four faces in both thalloid liverworts (Leitgeb, 1881; Kny, 1890) and hornworts (recently reviewed in Renzaglia, 1978). Extant sporophytes in all three bryophyte lineages exhibit entirely determinate development, developing from temporary ACs and/or intercalary basal meristems (reviewed in Bartlett, 1928; Crandall-Stotler, 1980; Kato and Akiyama, 2005). It is therefore possible that persistent ACs first arose in the gametophyte stage of the land plant lifecycle, and became incorporated into sporophyte development.

Whether a single AC truly represents the plesiomorphic state of the tracheophyte shoot apex is still debated (Banks, 2015; Harrison, 2015); a number of lycophyte and fern species posses multiple ACs at their apex (reviewed in White and Turner, 1995), whereas others such as the ferns *Nephrolepsis exaltata* (Sanders et al., 2011) and *C. richardii* (Hou and Hill, 2002) develop from a single tetrahedral AC (**Figure 3H**). Evidence from histology and clonal analysis suggests that *S. kraussiana* shoots develop from two adjacent ACs (**Figure 3J**; Harrison et al., 2007; Harrison and Langdale, 2010), although single ACs can be observed in the early stages of minor branch formation (Harrison et al., 2007) and other authors have suggested that this condition persists (Jones and Drinnan, 2009). The multicellular SAM in seed plant shoots is at least superficially more complex in structure than these examples. The SAM comprises discrete functional zones, namely a central multicellular zone of pluripotent cells and a surrounding peripheral zone of cells from which lateral organ primordia are specified; both zones overlap distinct tissue layers derived from separate cell lineages (reviewed in Gaillochet and Lohmann, 2015). It has recently been proposed that lycophyte and monilophyte ACs, subtended by a transcriptionally distinct and rapidly proliferating ‘core domain’ of daughter cells, might be functionally equivalent to the central zone of the SAM (Frank et al., 2015). Laser capture microdissection (LCM)-RNAseq comparison between apices from *S. moellendorffii*, the monilophyte *Equisetum arvense* and the angiosperm *Zea mays* (maize) found disparate expression profiles between the lycophyte and monilophyte ACs, but the core domain of both species expressed numerous genes associated with developmental regulation in the maize SAM (Frank et al., 2015). As such, envisaging the AC alone as functionally equivalent to a SAM may be too simplistic.

**FIGURE 4:**
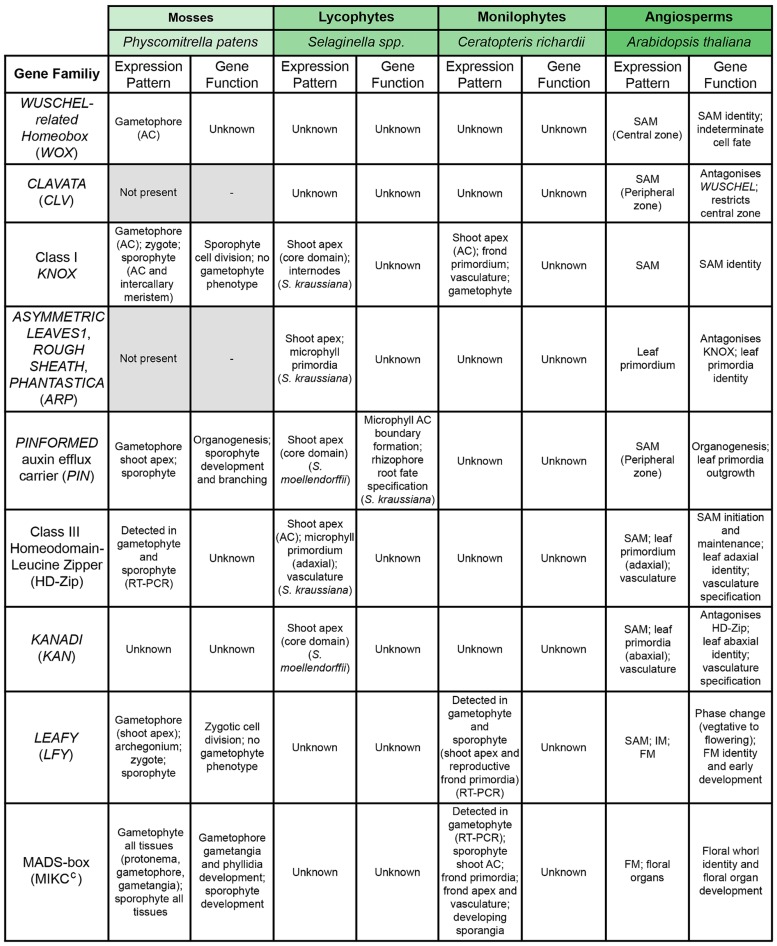
**Conservation of the genetic regulators of shoot apical meristem (SAM) function across model land plant species**. Summary table comparing known data about expression patterns and gene function of homologs of important regulators of the *A. thaliana* SAM across different land plant model species. Higher phylogenetic relationships between the model species are indicated by color coding (see **Figures 1** and **2**). In the case of *Selaginella*, genetic and developmental data come from both *S. kraussiana* and *S. moellendorffii*, as specified. Gene families highlighted in gray are absent from the genome of that particular species.

Sufficient data is now available to examine how homologs of genes with important functions in the *A. thaliana* SAM function in other land plant groups. A number of distinct modules are crucial to maintaining SAM identity and indeterminacy (summarized in **Figure 4**). The CLAVATA/WUSCHEL (CLV/WUS) pathway regulates the size of the apical initial domain within the multicellular SAM (Schoof et al., 2000; Bäurle and Laux, 2005), and the Class I KNOTTED1-like HOMEOBOX/ASYMMETRIC LEAVES, ROUGH SHEATH, PHANTASTICA (KNOX/ARP) pathway regulates indeterminate cell fate versus specification of the determinate leaf development program (Schneeberger et al., 1998; Timmermans et al., 1999; Tsiantis et al., 1999; Byrne et al., 2000; Guo et al., 2008; reviewed in Gaillochet and Lohmann, 2015). In some angiosperms Class 1 KNOX expression is later reactivated in established leaf primordia to generate compound leaves (see Phase Change section). Both the CLV/WUS and KNOX/ARP pathways require intercellular communication, which is mediated at least in part by movement of the component proteins between cells (Lucas et al., 1995; Lenhard and Laux, 2003; Yadav et al., 2011). A third family of transcription factors, Class III homeodomain-leucine zipper (HD-Zip), is also required for SAM formation and maintenance, the function of which is antagonized by the *KANADI* (*KAN*) genes (Emery et al., 2003; reviewed in Floyd and Bowman, 2007).

Although a *WUSCHEL-related HOMEOBOX* (WOX) gene is preferentially expressed in the *P. patens* gametophyte AC (Frank and Scanlon, 2015), the *CLV1* and *CLV2* gene families are absent from the *P. patens* genome (Banks et al., 2011), precluding the existence of the WUS-CLV regulatory module. In contrast, Class III HD-Zip and KAN homologs have been identified in *P. patens* (Sakakibara et al., 2001; Floyd and Bowman, 2007; Banks et al., 2011) but none are enriched in the AC (Frank and Scanlon, 2015). The expression of Class I *KNOX* genes has been reported in the *P. patens* gametophore AC (Frank and Scanlon, 2015) but no loss-of-function mutant phenotypes have been detected in the gametophyte (Singer and Ashton, 2007; Sakakibara et al., 2008), and *ARP* genes are absent from the *P. patens* genome (Banks et al., 2011). As such, there is currently no molecular evidence to support the suggestion that these components of the SAM regulatory network were established in gametophyte shoots.

The ancestral role for KNOX proteins is sporophytic, as inferred from studies in chlorophyte algae where hetero-dimerization of a KNOX and a BELLRINGER protein facilitates zygote formation (Lee et al., 2008). In angiosperms, Class 1 *KNOX* expression is essential for SAM maintenance (Long et al., 1996; Vollbrecht et al., 2000; Belles-Boix et al., 2006). In the *P. patens* sporophyte, Class 1 *KNOX* genes are expressed transiently in the AC during phases of cell proliferation. However, loss-of-function mutants demonstrated that although Class I *KNOX* genes promote sporophytic cell divisions and regulate their orientation, they are not essential for apical activity *per se* (Sakakibara et al., 2008). Within the tracheophytes, Class 1 *KNOX* expression has been detected in the shoot apices of both *S. kraussiana* and *S. moellendorffii* with expression localized to either the AC (Frank et al., 2015) or cells immediately subtending it (Harrison et al., 2005). In both cases, transcripts were absent from newly developing organ primordia, a pattern similar to that seen in the angiosperm SAM (e.g., Jackson et al., 1994). Class 1 KNOX activity in angiosperm leaf primordia is repressed by ARP gene function, as a consequence of which the two components display mutually exclusive expression patterns (Timmermans et al., 1999; Tsiantis et al., 1999; Byrne et al., 2000; Guo et al., 2008). Class 1 *KNOX* expression is seen in both the shoot apex and young organ primordia of the ferns *Osmunda regalis* (Harrison et al., 2005), *Annogramma chaeophylla* (Bharathan et al., 2002) and *C. richardii* (Sano et al., 2005), although transcripts are absent from older primordia. ARP homologs are expressed in the organ primordia of both *S. kraussiana* and *O. regalis* (older primordia only), but they are also co-expressed with Class 1 *KNOX* at the shoot apex (Harrison et al., 2005). The ancestral function of Class 1 KNOX appears to relate to cell division in the land plant sporophyte, but in the absence of mutant phenotypes in lycophytes or monilophytes it is impossible to say at what stage it became essential for AC/meristem maintenance. Based on observed expression patterns, the evolution of the mutually exclusive *KNOX*/*ARP* expression pattern may have occurred coincident with the formation of the SAM in seed plants. Overexpression and complementation studies in *A. thaliana* suggest that Class 1 *KNOX* and *ARP* homologs from lycophytes and monilophytes, respectively, can provide some of the same functions as endogenous *A. thaliana* genes (Harrison et al., 2005; Sano et al., 2005), but these experiments are not informative about what these genes do in their native context. Currently there is no transgenic system available in a lycophyte species, but future functional studies in *C. richardii* should begin to resolve some of these functional questions.

At present, much less is known about the function and expression of other SAM gene homologs within non-seed vascular plants. Although one *WOX* gene is detected at the *S. moellendorffii* shoot apex, it is not expressed in the AC, instead transcripts accumulate within the core domain and in developing primordia (Frank et al., 2015). There may be greater conservation of HD-Zip function between lycophytes and angiosperms: a Class III HD-Zip homolog is strongly expressed in the *S. kraussiana* shoot AC (Floyd et al., 2006) and *KAN* expression is up-regulated in the core domain beneath them (Frank et al., 2015). In the *C. richardii* sporophyte, expression of Class III HD-Zip homologs has been detected (Aso et al., 1999; Floyd et al., 2006) but spatial expression data is not yet available. Thus, although known regulators may have a role in apical development within vascular plants, whether their specific functions are conserved or differ remains to be established.

Phytohormones are another important regulatory force within the angiosperm SAM, acting to integrate other developmental signals. Cytokinin (CK) maintains the indeterminate central zone by promoting *WUS* expression in a complex multiple feedback loop (reviewed in Gaillochet and Lohmann, 2015), and CK biosynthesis is up-regulated by the Class 1 *KNOX* gene *SHOOTMERISTEMLESS* (*STM*; Jasinski et al., 2005; Yanai et al., 2005), thus linking Class 1 KNOX and WUS activity. STM also represses biosynthesis of gibberellin (GA) within the SAM (Jasinski et al., 2005), which otherwise promotes tissue growth and differentiation. Interestingly, a homolog of the GA response-repressing DELLA transcription factor is up-regulated in the *S. moellendorffii* shoot apex (Frank et al., 2015), suggesting a conserved requirement for GA suppression, although the same was not found in the monilophyte *E. arvense*. In *P. patens*, CK signaling-related transcripts are up-regulated in the gametophore AC (Frank and Scanlon, 2015), and exogenous CK promotes AC identity, causing increased branching and the development of ectopic meristematic cells in callus-like tissue (Coudert et al., 2015). Application of CK is also sufficient to induce callus tissue at the shoot apex of *C. richardii* sporophytes (Plackett et al., 2014). Collectively these observations point to an ancestral and conserved function for CK in regulating AC function and shoot development. Importantly, loss of Class 1 KNOX function does not perturb the expression of CK biosynthesis gene homologs in the *P. patens* sporophyte (Sakakibara et al., 2008), indicating that functional links between Class 1 KNOX and CK emerged in the tracheophyte lineage.

A second hormone, auxin, functions to promote pluripotency in the central zone of the angiosperm SAM by enhancing CK signaling (reviewed in Gaillochet and Lohmann, 2015), and both CK and auxin signaling are present in all land plant lineages (Wang et al., 2015). Disruption to polar auxin transport (PAT) in *S. kraussiana* causes the shoot apex to terminate, supporting a conserved function for auxin in indeterminate cell fate in lycophyte and angiosperm shoot apices (Sanders and Langdale, 2013). Notably, *in situ* analysis of a PIN auxin transporter in *S. moellendorffii* detected *PIN* expression surrounding the shoot AC, with a concomitant increase in expression of an *AUXIN RESPONSE FACTOR* (*ARF*) in the AC (Frank et al., 2015). This suggests the presence of an auxin maximum (peak in concentration) at the lycophyte AC. Presumably perturbed PAT therefore leads to a decrease in auxin levels in the AC, and hence to the observed termination (Sanders and Langdale, 2013). Recent analysis in *M. polymorpha* also found the greatest concentration of auxin in apical/meristematic regions (Eklund et al., 2015). Conversely, phenotypic analysis of *pin* mutants in *P. patens* identified a role for PAT in maintaining gametophore AC function by preventing auxin accumulation at the AC (Bennett et al., 2014). This apparent contradiction may relate to the inhibitory role of auxin in suppressing axillary branching in the moss gametophore, which is not found in lycophytes or monilophytes (see Shoot Branching section). Despite this difference, interactions between the auxin and CK signaling pathways are thought to promote AC fate in the *P. patens* gametophore (reviewed in Kofuji and Hasebe, 2014), as they do in angiosperm SAMs. As such, the auxin-CK signaling module likely became associated with AC function and shoot indeterminacy in the earliest diverging land plants.

## Organogenesis and Lateral Organ Development

It is often assumed that the morphology of the majority of extant bryophyte sporophytes is representative of ancestral sporophytes, being single axes with no lateral outgrowths (reviewed in Kato and Akiyama, 2005). It has been proposed that the transition from an unbranched shoot axis to a complex, indeterminate shoot branching system occurred in a stepwise fashion (reviewed in Tomescu et al., 2014). One of the most significant steps in this trajectory was the evolution of lateral organs, i.e., ‘leaves.’ Leafless fossils have been assigned to each of the lycophyte, monilophyte, and seed plant clades (Kenrick and Crane, 1997), and subsequent analysis of fossil characters against extant species strongly suggest that lateral organs evolved independently within the three tracheophyte lineages (Boyce and Knoll, 2002; Sanders et al., 2009; Tomescu, 2009). To avoid confusion, the term ‘megaphylls’ (describing both fern fronds and seed plant leaves) is not used in this review because of their probable independent origins. The term ‘frond’ is instead used to distinguish monilophyte lateral organs from the seed plant ‘leaf.’ Importantly for developmental studies, it has been proposed that lycophyte lateral organs (‘microphylls’) have an independent evolutionary origin to both fronds and leaves, arising as tissue outgrowths which later became vascularized (the enation theory; Bower, 1935) as opposed to being modified lateral branches of vascularized shoots (the telome theory; Zimmermann, 1952). Notably, a comparison of genetic mechanisms operating in these different lateral organs provided evidence for KNOX/ARP function in the formation of microphylls, monilophyte fronds and seed plant leaves (Harrison et al., 2005). This suggests that the same pathway was recruited to distinguish determinate lateral organs from indeterminate shoots during the evolution of both microphylls and megaphylls.

Lateral organs arise sequentially from the shoot apex across all lineages, but through different generative mechanisms. Angiosperm lateral organ primordia develop from multicellular populations of founder cells specified at the periphery of the SAM whereas lateral organs in non-seed plant lineages arise from a single or a few initials derived from the shoot AC (reviewed in Steeves and Sussex, 1989; Gaillochet et al., 2015). Lateral organ ACs are for the most part morphologically distinct from their corresponding shoot AC (with the exception of *S. kraussiana*), comprising wedge or lenticular shapes with only two cutting faces (**Figure 3K**). Similar AC shapes are found in early stages of bryophyte thallus development (reviewed in Ligrone et al., 2012). The phyllidia of moss gametophores each arise from a single AC that is specified within two cell divisions of the shoot AC (Harrison et al., 2009). Sector analysis demonstrated that microphylls arising from *S. kraussiana* shoots initiate from a pair of adjacent ACs (Harrison et al., 2007), strikingly mirroring the two-celled nature of the shoot apex. Fern fronds typically (but not always) arise from a single AC (reviewed in Vasco et al., 2013). Non-seed plant lateral organ growth is therefore largely driven by ordered patterns of cell division at the tip of the structure (**Figure 3**), whereas in seed plant leaves cell divisions occur across the organ and morphogenesis is co-ordinated by non-cell autonomous ‘supracellular’ mechanisms (reviewed in Dengler and Tsukaya, 2001; Fleming, 2002).

Whilst a great deal is now understood about the specification of angiosperm leaf primordia, in which positional signals such as transient auxin maxima are critical (see below), very little is known about the specification of lateral organ initials. In both *P. patens* gametophores (Harrison et al., 2009) and *S. kraussiana* shoots (Harrison et al., 2007), cells arising from shoot and leaf initials follow predictable fates. Although these patterns could indicate cell-autonomous mechanisms for specification, it has been demonstrated in similarly predictable systems that perturbations to division patterns do not change cell fate specification (e.g., van den Berg et al., 1995, 1997), and thus that non-cell autonomous signals can at least compensate for loss of any lineage-based mechanisms. Studies of fern development have found that new frond and pinna initials are specified within distinct merophytes, i.e., groups of related cells descended from a single daughter cell of the AC, in a manner similar to that seen in *P. patens* and *S. kraussiana* (Hou and Hill, 2002; Sanders et al., 2011). However, a role for non-cell autonomouss signals is more evident in this case because newly arisen frond primordia develop as shoots if grown in isolation from the shoot apex and older fronds, demonstrating that frond identity is specified by the apex and/or other fronds (reviewed in Vasco et al., 2013). In *C. richardii*, specification of the frond initial is increasingly delayed after cleavage from the shoot AC as development progresses (Hou and Hill, 2002), and patterning of sporangia on reproductive pinnae is dependent on cell position (Hill, 2001). Thus, although fern frond development displays tip-based acropetal growth in common with bryophytes and lycophytes, there is also evidence for non cell-autonomous regulation of developmental patterning in common with angiosperms.

In the case of bryophytes, lycophytes, and seed plants, development of individual lateral organs is determinate. In contrast, fern frond development is iterative, with further subordinate ACs arising from the products of the frond AC, resulting in the outgrowth of pinnae (**Figures 3C–E**). Interestingly, pinna development on *C. richardii* reproductive fronds is driven by the activity of two adjacent ACs (**Figure 3J**; Hill, 2001), rather than the single AC seen at the apex of vegetative fronds (**Figure 3I**; Hou and Hill, 2002), suggesting a functional distinction between the two hierarchical levels (**Figure 3K**). Frond development is fully indeterminate in some fern species (Vasco et al., 2013), and fossil fronds of early monilophytes also contain indeterminate characters (Sanders et al., 2009). Together with fossil analysis that shows shoot branching in both fern and seed plant lineages prior to the emergence of lateral organs (Sanders et al., 2009), these observations suggest that fern fronds originated as modified shoots.

Polar auxin transport is an essential component of organogenesis at the angiosperm SAM (Reinhardt et al., 2003), where transient auxin maxima in the peripheral zone specify the site of each incipient lateral organ (Vernoux et al., 2011). Similarly, blocking PAT in the *P. patens* gametophore disrupts phyllidia outgrowth and development (Bennett et al., 2014; Viaene et al., 2014), with extreme examples lacking lateral organs entirely. PAT is also necessary for correct boundary formation between the shoot ACs and microphyll initials in *S. kraussiana*, but microphyll initiation *per se* is unaffected by inhibition of PAT (Sanders and Langdale, 2013). The functions of PAT in the fern sporophyte remain to be investigated, but microsurgical experiments found that primordia do not arise independently, in that each primordium influences the positioning of subsequent primordia at the shoot apex (reviewed in Vasco et al., 2013). At least superficially, this reflects what happens in the angiosperm shoot apex. These observations suggest a conserved role for auxin and PAT in specifying which cells at the apex contribute to lateral organs, and a more divergent role in organ initiation and outgrowth. PAT also has a conserved role in specifying which cells within the lateral organ will form vascular tissue, influencing venation patterns in angiosperm leaves (Scarpella et al., 2006), fronds of the fern *Matteucia struthiopteris* (Ma and Steeves, 1992) and microphylls of *S. kraussiana* (Sanders and Langdale, 2013). More detailed analysis of auxin and PAT function in *C. richardii* shoot development would determine the extent to which these different auxin functions are each conserved within the vascular plants.

As in the case of auxin, two aspects of HD-Zip function appear to be differentially conserved in vascular plants. In addition to functions within the SAM, Class III HD-Zip transcription factors in *A. thaliana* specify adaxial fate and sites of vascular development in newly formed leaf primordia (Prigge et al., 2005; reviewed in Floyd and Bowman, 2007). Expression patterns in two gymnosperms indicate a conserved role for specifying adaxial leaf fate in seed plants (Floyd and Bowman, 2006), but no expression is found in newly formed microphyll primordia of *S. kraussiana* (Floyd and Bowman, 2006). In contrast, expression patterns support a conserved role in the developing vasculature of *S. kraussiana* microphylls (Floyd and Bowman, 2006; Floyd et al., 2006). Given that vasculature evolved in the tracheophytes prior to lateral organs, it is likely that HD-Zips and auxin were first recruited to specify veins, a role that is conserved in extant lycophytes, ferns, and seed plants. When lateral organs subsequently evolved in each of the three lineages, HD-Zip function was modified for specification of leaf polarity in seed plants but not in lycophytes. Closer analysis of HD-Zips in fern frond development would determine whether a role in leaf polarity was independently adopted in monilophytes, and functional analysis in *P. patens* should reveal the ancestral role in non-vascular plants.

## Shoot Branching

The ability to branch is a key innovation in sporophyte shoot development. Two distinct branching systems are found in tracheophytes: apical branching, where the shoot apex bifurcates; and the outgrowth of lateral (axillary) meristems produced in association with lateral organs (reviewed in Sussex and Kerk, 2001). Apical branching is found across the tracheophytes, including lycophytes (such as *S. kraussiana*; Harrison et al., 2007), monilophytes (although not *C. richardii*; Bierhorst, 1977) and in some seed plants (reviewed in Gola, 2014). The existence of tracheophyte fossils such as *Cooksonia*, which have determinate, branched sporophytes (reviewed in Boyce, 2010), suggests that apical branching is the ancestral sporophytic branching mechanism. In addition, the existence of non-vascular polysporangiate fossils (recently reviewed in Edwards et al., 2014) indicates that sporophyte branching emerged prior to the divergence of the first tracheophytes. Most extant bryophyte sporophytes comprise a single axis, but examples of sporophyte apical branching have also been reported in extant mosses and liverworts (Leitgeb, 1876; Györffy, 1929; Bower, 1935). Apical branching is also seen during thallus development of liverwort and hornwort gametophytes (Schuster, 1984a,b). Thus the capacity for sporophyte branching presumably first originated prior to the divergence of bryophytes and vascular plants, but whether branched sporophytes represent an ancestral state in any of the bryophyte lineages is unknown.

Multiple different cellular mechanisms for apical branching have been described across land plants (reviewed in Gola, 2014), such as the proliferation of existing ACs in *S. kraussiana* to establish new axes without interruption (Harrison et al., 2007) or the loss of a single AC followed by initiation of multiple new branch initials, as seen in some leptosporangiate ferns (Hébant-Mauri, 1993). The genetic mechanisms underlying apical branching are poorly understood. Experiments in *P. patens* demonstrated that disturbance of PAT or *LEAFY* (*LFY*) gene function can induce sporophyte branching and the production of two terminal sporangia (Tanahashi et al., 2005; Fujita et al., 2008; Bennett et al., 2014). In *S. kraussiana* and fern shoot apices, branching occurs in a regular pattern after a fixed number of lateral organs have been initiated (Bierhorst, 1977; Harrison et al., 2007), suggesting the involvement of a time or distance-dependent regulatory mechanism. Excising *S. kraussiana* shoot tips from a parent plant disrupts this mechanism, resulting in a far greater interval before branching re-initiates, and thus implying that branching is regulated by a mobile signal (Sanders and Langdale, 2013). Auxin is an important branching regulator in seed plants, imposing apical dominance by inhibiting outgrowth of axillary buds through basipetal PAT (reviewed in Müller and Leyser, 2011). *S. kraussiana* shoots exhibit basipetal PAT, but inhibiting auxin transport did not affect the branching interval (Sanders and Langdale, 2013). The different apical branching modes could reflect either convergent evolution of different mechanisms or subsequent diversification from an ancestral branching mechanism. Further study in all non-seed plant lineages is necessary to resolve this.

Early tracheophyte sporophyte fossils exhibit equal (dichotomous) branching (Boyce, 2010), but in subsequent lineages shoot architecture is more complex, with unequal branch growth and apical dominance. Apical branching in *S. kraussiana* is unequal (**Figure 3B**): one branch becomes the major growth axis because of an unequal partitioning of the AC population at the time of branching (Harrison et al., 2007). Unequal branch growth has been proposed as an important component in the origins of lateral organs as part of the telome theory (reviewed in Sussex and Kerk, 2001), with a progression from equal (dichotomous) branching to an asymmetric branching structure with a dominant shoot apex. The regulatory mechanisms underpinning the evolution of unequal growth have so far not been investigated. Shoot growth in seed plants is regulated by the hormone GA (reviewed in Fleet and Sun, 2005), triggering degradation of the DELLA transcription factors that otherwise restrict growth (reviewed in Ueguchi-Tanaka and Matsuoka, 2010). Functional GA signaling evolved after the divergence of the bryophytes (Hirano et al., 2007; Yasamura et al., 2007), although evidence from *S. moellendorffii* suggests that GA originally regulated reproductive development and not vegetative shoot growth (Aya et al., 2011). The advent of unequal branch growth in the tracheophyte sporophyte might therefore be linked with the co-option of GA signaling as a regulator of vegetative growth.

In contrast to other tracheophytes, shoot architecture in seed plants is dominated by axillary branching (reviewed in Sussex and Kerk, 2001). *De novo* lateral meristems arise in the axils of leaves after lateral organ formation, a process that requires a local depletion of auxin followed by a ‘pulse’ of CK (Wang et al., 2014). Basipetal PAT from the SAM inhibits axillary bud outgrowth by maintaining high local auxin concentrations, whereas CK promotes their activation by antagonizing auxin function (reviewed in Müller and Leyser, 2011). Axillary branching is also found in moss gametophores where lateral branches arise from single initials re-specified from epidermal cells (Berthier, 1972 and references therein; reviewed in La Farge-England, 1996). Experiments in *P. patens* show that, in striking similarity to seed plant shoots, apically synthesized auxin creates a zone of branching inhibition equivalent to apical dominance (although basipetal PAT is not involved), whilst CK correspondingly promotes branching (Coudert et al., 2015). The degree to which this represents convergent evolution is unclear. Chemical and genetic manipulation of auxin levels in *M. polymorpha* indicate a role in apical dominance and branching in the liverwort thallus (Kaul et al., 1962; Binns and Maravolo, 1972; Davidonis and Munroe, 1972; Maravolo, 1976; Flores-Sandoval et al., 2015), indicating a potential ancestral role for auxin in apical dominance at the base of the land plants. A third hormone, strigolactone (SL), is an important repressor of branch outgrowth in angiosperms (reviewed in Janssen et al., 2014). SL biosynthesis and signaling are thought to have originated prior to the evolution of land plants (Delaux et al., 2012; Wang et al., 2015), and SL has been shown to similarly repress branching in the *P. patens* gametophore (Coudert et al., 2015). Thus, the hormonal regulation of axillary branching is strongly similar in bryophyte gametophytes and seed plant sporophytes.

The precise origins of axillary branching remain unknown. Interestingly, branch points in *Selaginella* species generate *de novo* structures termed ‘angle meristems’ (Cusick, 1954; Jernstedt et al., 1992). These can develop into aerial roots or shoots, with shoot fate promoted through increased CK or inhibition of PAT (Sanders and Langdale, 2013). The nature of fern fronds is still not fully resolved, but they bear a superficial resemblance to the axillary shooting structure in seed plants, in that ACs are initiated at the frond margin in a hierarchical manner to produce pinnae (Sanders et al., 2011). Many fern species (including *C. richardii*) also develop *de novo* foliar buds on the adaxial surface of otherwise differentiated lateral organs, which are capable of becoming independent sporophytes (reviewed in Vasco et al., 2013). From these observations, it can be hypothesized that axillary branching in seed plants may have been derived from mechanisms of lateral apical development similar to that seen in fern fronds, potentially relating back to ancestral apical branching mechanisms. However, given that axillary buds are derived from the adaxial surface of the developing lateral organ primordia in angiosperms (McConnell and Barton, 1998), it is perhaps more likely that the axillary branching mechanisms were co-opted from those operating to form *de novo* shoots in the context of monilophyte foliar buds and/or lycophyte rhizophores. In either case, a greater understanding of fern shoot and frond development will be highly informative in addressing this question.

## Phase Change- Modifying Apical and Lateral Organ Development

It can be inferred from extant bryophytes that the ancestral sporophyte was purely reproductive in nature, consisting entirely of a stalked sporangium. In contrast, all tracheophyte sporophytes precede reproduction with a vegetative phase that can be short or prolonged depending on the combined activity of endogenous developmental cues and external environmental signals. The developmental origins of this vegetative phase are unclear, and theories to explain its appearance include sterilization of sporangia or interpolation of a novel vegetative structure prior to development of the ancestral reproductive sporophyte (reviewed in Tomescu et al., 2014). Expression analysis of embryonic liverwort and moss sporophytes found evidence of meiosis-associated gene function, even prior to visible sporangium formation (Frank and Scanlon, 2015). It has been proposed that repression of these genetic programs in the early sporophyte led to indeterminate development and, ultimately, the emergence of a vegetative phase. This evidence is consistent with the hypothesis of Bower (1908) that proposed that the elaboration of the sporophyte was driven by selective pressure to delay meiosis (recently reviewed in Qiu et al., 2012).

The vegetative phase can be further sub-divided into juvenile and adult phases, the transitions distinguishable in angiosperms through changes in leaf shape and properties such as leaf hairs and cuticle composition (reviewed in Huijser and Schmid, 2011). Similarly, *S. kraussiana* exhibits a developmental phase change, distinguishable as a change from juvenile spiral phyllotaxy to adult dorsiventral asymmetry (Harrison et al., 2007). Consistent with this, *C. richardii* fronds also undergo a strong and gradual heteroblastic change in morphology during vegetative development, progressing from simple, spade-shaped lamina to highly dissected forms (Hou and Hill, 2002). In angiosperms these phase changes, including the transition to reproductive development (see below) are regulated by two microRNAs, miR156 and miR172 (reviewed in Huijser and Schmid, 2011). miR156 expression has been detected in non-seed plants including mosses and ferns, although corresponding gene function is not known, whereas conservation of miR172 outside of the angiosperms is still subject to debate. Downstream of these regulators, changes in leaf shape between the juvenile and adult phases in angiosperms are caused by a number of diverse, independently originating mechanisms (reviewed in Bar and Ori, 2015), including reactivation of Class 1 *KNOX* gene expression in leaf primordia (e.g., in tomato; Hareven et al., 1996), ectopic expression of *LFY* (e.g., in pea; Hofer et al., 1997), or through the activity of the REDUCED COMPLEXITY (RCO) homeodomain protein (e.g., in b*rassicas*; Vlad et al., 2014). Activity of these transcription factors, along with the organization of discrete auxin maxima along the leaf margin, promote localized cell divisions in the leaf that convert entire leaf blades into more complex structures with serrated, dissected or compound morphology. Whether the underlying mechanisms driving changes in *C. richardii* frond morphology are conserved with those regulating phase transitions in seed plants is currently unknown.

The most evident phase change during tracheophyte shoot development is the transition to reproductive growth (reviewed in Huijser and Schmid, 2011). In angiosperms, the SAM converts to an inflorescence meristem (IM) that produces floral meristems (FMs) subtended by bracts at its periphery. This transition is promoted by the LFY transcription factor, which is up-regulated in the SAM (Weigel et al., 1992). LFY also plays a role in the reproductive transition in gymnosperms (Mouradov et al., 1998). In *P. patens*, however, LFY instead regulates the first division of the zygote (Tanahashi et al., 2005), a function clearly distinct from its known role in seed plants. *P. patens LFY* (*PpLFY*) homologs are expressed in gametophore shoot apices throughout development, and also in the developing archegonium and the developing sporophyte (Tanahashi et al., 2005). However, no loss-of-function mutant phenotype is obvious in the gametophyte. *C. richardii LFY* (*CrLFY*) homologs are also expressed in both gametophytic and sporophytic tissues (Himi et al., 2001), but no functional data have yet been reported. These observations suggest ancestral functions for the *LFY* gene family in tracheophytes that cannot be predicted from our current knowledge of seed plants.

Whether LFY has a role in reproductive transitions in lycophytes and monilophytes is not known, but this question is particularly pertinent in ferns where sporangia develop on ‘fertile’ fronds rather than as distinct structures arising from the shoot apex. Transcriptional analysis of the fern *A. filiculoides* found that homologs of genes associated with the angiosperm floral transition were up-regulated in sporogenous tissues, including *FLOWERING TIME* (*FT*) and *LFY* (Brouwer et al., 2014). Consistent with a role in reproductive development, *CrLFY* expression has also been detected in the shoot apex and in developing reproductive fronds (Himi et al., 2001). The functional divergence of LFY between angiosperms and bryophytes is reflected in changes in protein structure that alter target specificity (Sayou et al., 2014). As a consequence, *PpLFY* homologs cannot complement *lfy* loss-of-function mutants in *A. thaliana* (Maizel et al., 2005). A *CrLFY* homolog (*CrLFY2*) can partially complement the *A. thaliana lfy* mutant (Maizel et al., 2005), indicating some functional conservation but equally that CrLFY function is not identical to that in angiosperms. Notably, *LFY* expression in angiosperms is promoted by GA to induce flowering (Blázquez et al., 1998) and GA treatment of *Ceratopteris thalictroides* accelerates the production of fertile fronds (Stein, 1971). These results suggest that at least some reproductive functions of *LFY* might be conserved between monilophytes and seed plants, although in light of changing target specificity the downstream mechanisms could vary (see below).

Floral meristem development represents a modification of organogenesis and shoot development within the angiosperms, producing modified lateral (floral) organs in successive, concentric whorls and then terminating the meristem (reviewed in Irish, 2010). Most closely studied in *A. thaliana*, floral organ identity and shoot determinacy are governed by MADS box transcription factors. Two major types of MADS box genes are found across eukaryotes, with type I MADS box genes having received least attention. Type I genes are involved in female gametophyte development and post-zygotic lethality of interspecific hybrids, and mutants generally display phenotypes that are only subtly different from wild-type (Alvarez-Buylla et al., 2000). Type II MADS box genes, with a role in gamete formation in representatives of the sister lineage to land plants (Tanabe et al., 2005), underwent a duplication after the transition to land, diverging into an MIKC* clade, implicated mainly in male gametophyte development (Zobell et al., 2010; Kwantes et al., 2012) and an MIKC^c^ clade, functioning mostly in the sporophyte. In seed plants, MIKC^c^ MADS box genes are expressed, with one exception, exclusively in the sporophyte generation (Zobell et al., 2010), whereas they are expressed in both generations in ferns and mosses (Münster et al., 1997; Hasebe et al., 1998; Quodt et al., 2007). It is therefore presumed that MIKC^c^ MADS box gene function became canalized from an ancestral role in gametophyte development to sporophyte reproduction in seed plants (Nishiyama et al., 2003).

In a revealing analogy to how HOX genes organize the animal body plan (Shubin et al., 1997), floral MIKC^c^ MADS box genes were first described in angiosperms for their patterning role during flower development, as part of the ABCE model (Bowman et al., 1989; Schwarz-Sommer et al., 1990; Coen and Meyerowitz, 1991; Pelaz et al., 2000). The eye-catching examples whereby homeotic mutations swapped organs such as legs and antenna in the fly *Drosophila melanogaster* or petals and stamens in *A. thaliana* flowers propelled and popularized the field of evo-devo. But beyond the in-depth studies conducted originally in the model flowers of *A. thaliana* and *Antirrhinum majus*, and the necessary modifications to the ABCE model when delving into other branches of the flowering plant phylogeny (Litt and Kramer, 2010), little is known today about the function of ancestral MADS box genes, prior to the evolution of seed plants.

Interrogation of the *S. moellendorffii* genome and subsequent analyses conclude that at least two Type II genes were present in the common ancestor of vascular plants (Banks et al., 2011; Gramzow et al., 2012) but the ABCE class genes are seed plant-specific. Gymnosperms have orthologs of B and C class MIKC^c^ genes; the expression of the former during male cone development and the later during female and male cone development, points to a conserved role in sporophyte reproductive structures across seed plants (Tandre et al., 1995, 1998; Rutledge et al., 1998; Mouradov et al., 1999; Shindo et al., 1999; Sundström et al., 1999; Winter et al., 1999; Jager et al., 2003; Zhang et al., 2004). The origin of the different clades of angiosperm-specific MIKC^c^ genes, including the floral homeotic genes, is presumed to trace back to the seed plant ancestor after the evolution of ferns (Gramzow and Theißen, 2015). This hypothesis is consistent with the absence of floral homeotic gene orthologs from the genomes of *P. patens* and *S. moellendorffii* (Rensing et al., 2008; Banks et al., 2011; Gramzow et al., 2012). Similarly, homologs of MIKC^c^ genes identified from ferns cannot be assigned to particular subclades of ABCE class genes. In *C. richardii*, at least eight MIKC^c^ MADS box genes belonging to three main clades have been reported, representing an independent line of evolution from the seed plant MADS box genes and occupying an intermediate position between those of moss and of the major clades of seed plants (Münster et al., 1997; Hasebe et al., 1998; Gramzow et al., 2012; Kwantes et al., 2012). Expression of these genes has been detected in the shoot apex and in both developing vegetative and reproductive fronds (**Figure 4**; Hasebe et al., 1998). Within the bryophytes, *P. patens* has six MIKC^c^-type MADS box genes, which are expressed in both the gametophyte and sporophyte generation, and development of both is impaired upon down-regulation (Quodt et al., 2007; Singer et al., 2007).

In light of the documented substantial bias toward seed plants (mostly angiosperms) in the study of MIKC^c^ MADS box genes, the prospect of investigating the role of these important regulators of plant development in an evolutionary intermediate plant lineage such as ferns is timely. Unfortunately, the current lack of a transgenic system in lycophytes hinders any immediate prospects of learning about their function in the earliest lineage of vascular plants. Yet the time is ripe for functional studies in a representative of the fern lineage. Such studies will offer a unique glimpse into the function of these genes before they evolved their important role in the development of the flower as a key innovation.

Within the angiosperms the MIKC^c^ MADS box genes are directly activated by LFY (reviewed in Irish, 2010). While the regulatory relationship between *LFY* and the floral homeotic genes is an established fact in angiosperms, and possibly conserved within seed plants, it is unknown how early in land plant evolution this module was established. Coincident patterns of gene expression between the gymnosperm *LFY* homolog *NEEDLY* (*NDLY)* and the MIKC^c^ MADS box genes, and the ability of *NDLY* to largely rescue the *A. thaliana* mutant *lfy-1* suggested that LFY-mediated regulation of floral MADS box orthologs was already present in the ancestor of seed plants (Mouradov et al., 1998). Whether *LFY* regulates MADS box genes outside of seed plants is unclear. Non-overlapping patterns of expression of *C. richardii LFY* and MADS box genes suggest that *LFY* homologs may not have functioned as regulators of MIKC^c^ MADS box genes prior to seed plants (Münster et al., 1997; Hasebe et al., 1998; Himi et al., 2001). Functional analysis of *CrLFY* in *C. richardii* will facilitate the reconstruction of the *LFY* gene response network in general, and of its relationship to the MADS box genes in particular.

## Conclusion and Perspectives

In seed plants, shoots and organs develop from the co-ordinated activity of multiple cells, requiring complex intercellular communication to co-ordinate development. In contrast, non-seed plant shoots typically develop from single or multiple distinct ACs. With little known about the genetic pathways underlying AC function, based on morphology they were considered to be functionally divergent from the SAM. However, recent cell-specific expression analysis has suggested that it is inappropriate to think of ACs as single-cell shoot apices, with a number of regulatory mechanisms associated with seed plant SAMs expressed in tissues immediately surrounding them. A comparison between ACs from different land plant lineages suggests a gradual accumulation of conserved shoot apical regulatory mechanisms, a number of which (e.g., Class 1 KNOX, CK and auxin) are associated with apical function in earliest diverging bryophyte lineages. Beyond the shoot apex, however, greater divergence is evident in the regulation of organogenesis and subsequent lateral organ development. This is perhaps to be expected, as it reflects the independent origins of lateral organs within each lineage of vascular plants: the lycophytes, monilophytes, and seed plants.

Close scrutiny of the monilophyte (fern) shoot system highlights differences compared to both the moss and flowering plant developmental models, *P. patens* and *A. thaliana*. Arising from single or paired ACs, *C. richardii* shoot and frond development nevertheless shows indications of complex supracellular regulation more similar to flowering plants than to moss. However, the evidence to date points to independent origins for fern fronds and seed plant leaves, with frond development more equivalent to flowering plant shoots than to leaves. In short, ferns are not more elaborate mosses or slightly simpler flowering plants, but posses a distinct and complex developmental identity of their own.

In most aspects of shoot development covered in this review, relatively clear trajectories can be inferred between the bryophytes and the tracheophytes, including the conservation or adaptation of several ancestral regulatory mechanisms. Although major evolutionary changes occured during the bryophyte-tracheophyte transition, equally significant alterations to shoot development occurred within the vascular plant lineages, and the questions regarding these are often intractable based on current data. As sister group to the seed plants, exploring fern development has the potential to dramatically improve our understanding of seed plant evolution and to fully resolve the broader evolutionary trajectories that have occurred in land plants as a whole. Throughout this review we have highlighted numerous specific examples where further information regarding gene function in a fern would be invaluable. Given the broad diversity of the monilophytes, it is probable that numerous model species will ultimately be required from within this clade to fully understand different adaptive aspects of their development. Despite its derived aquatic adaptations, *C. richardii* can be considered a good candidate as an initial model species because, as a leptosporangiate fern, it represents a major clade within the monilophytes. Of crucial advantage, however, are the facts that it has already been established for laboratory use and the tools for genetic analysis in this species have now been developed. Forward genetic analysis (i.e., identification of unknown genes involved in a particular developmental process through mutants) has been exploited successfully in *C. richardii* to elucidate the pathway regulating sex-determination during gametophyte development (Warne and Hickok, 1991; Banks, 1994, 1997; Strain et al., 2001), and a double-haploid mapping population between two *C. richardii* ecotypes was used to create a genetic linkage map (Nakazato et al., 2006). Until now, however, such approaches have been severely limited by a lack of resources, with large-scale mutant libraries such as those available for *A. thaliana* not yet established. The recent development of methods to genetically transform *C. richardii* is therefore an important milestone, as it will allow investigation of gene function in a monilophyte via a reverse genetics approach (manipulation of candidate gene expression or function), and hopefully also provide the impetus to improve the other genetic resources available for this and other fern species.

## Conflict of Interest Statement

The authors declare that the research was conducted in the absence of any commercial or financial relationships that could be construed as a potential conflict of interest.
